# Palliative care in policy documents for adults with cancer and non-cancer diseases with potential palliative care needs: a document analysis

**DOI:** 10.1177/26323524241296145

**Published:** 2024-12-03

**Authors:** Anna O’Sullivan, Linnéa Carling, Joakim Öhlén, Stina Nyblom, Anneli Ozanne, Ragnhild Hedman, Carl-Johan Fürst, Cecilia Larsdotter

**Affiliations:** Department of Nursing Science, Sophiahemmet University, P.O. Box 5605, Stockholm SE-114 86, Sweden; Department of Health Care Sciences, Marie Cederschiöld University, Stockholm, Sweden; Department of Palliative Care, Sahlgrenska University Hospital, Gothenburg, Sweden; Institute of Health and Care Sciences, University of Gothenburg, Gothenburg, Sweden; Institute of Health and Care Sciences, Sahlgrenska Academy, University of Gothenburg, Gothenburg, Sweden; Centre for Person-Centred Care (GPCC), University of Gothenburg, Gothenburg, Sweden; Palliative Centre, Sahlgrenska University Hospital, Gothenburg, Sweden; Palliative Centre, Sahlgrenska University Hospital, Gothenburg, Sweden; Sahlgrenska Academy, Institute of Medicine, University of Gothenburg, Gothenburg, Sweden; Institute of Health and Care Sciences, Sahlgrenska Academy, University of Gothenburg, Gothenburg, Sweden; Department of Neurology, Sahlgrenska University Hospital, Gothenburg, Sweden; Department of Nursing Science, Sophiahemmet University, Stockholm, Sweden; Faculty of Medicine, Department of Clinical Sciences, Lund and The Institute for Palliative Care, Respiratory Medicine, Allergology and Palliative Medicine, Lund University, Lund, Sweden; Department of Nursing Science, Sophiahemmet University, Stockholm, Sweden

**Keywords:** cancer, chronic disease, end-of-life care, document analysis, palliative care, policy

## Abstract

**Background:**

The inclusion of palliative care in policy has been encouraged internationally, and gradually implemented, including in Sweden. Care should be driven by policy; hence, examining how palliative care is included in national policy documents is paramount.

**Objectives:**

This study aimed to examine how palliative care is included in national disease-specific policy documents for adults with chronic conditions, cancer and non-cancer, with potential palliative care needs.

**Design:**

Document analysis.

**Methods:**

A document analysis of Swedish policy documents for different disease-specific groups with severe chronic conditions, cancer and non-cancer, was performed. In total, 96 documents were analysed.

**Results:**

How palliative care was included in the policy documents varied from mentioning the term without explanation to detailed discussion regarding palliative care practice. Such discussion encompassed several conceptualisations of palliative care: defined through authorities’ definitions; as care of dying persons; integrated with disease-specific care and treatment; limited to disease-specific medical treatments or based on detail regarding certain key elements of palliative care such as specialised palliative care and end-of-life conversations.

**Conclusion:**

There may be large variations in how palliative care is conceptualised in national disease-specific policy documents, as disclosed by this analysis of the Swedish case. Limiting palliative care to disease-specific medical treatments (most commonly palliative oncological treatments) or the care of dying persons limits its scope in ways contrary to current evidence supporting early integrated palliative care. The lack of palliative care recommendations adapted for each specific diagnosis indicates a gap in policy. To promote equal access to palliative care regardless of patients’ diseases or medical conditions, the importance of how palliative care is included in national policy documents needs to be further acknowledged and discussed – with palliative care consistently included in such documents.

## Background

Palliative care needs are predicted to increase internationally over the coming decades, due to better living conditions and more advanced treatments resulting in the ageing of populations worldwide, with a high prevalence of life-limiting chronic illness,^1,2^ and prolonged life expectancy has increased over the years.^3^ It has been estimated that approximately 74% of the worldwide deceased population had potential palliative care needs.^2^ Nearly a decade ago, the World Health Organization (WHO) encouraged member states to develop and implement palliative care policies to strengthen health systems by integrating evidence-based, cost-effective and equitable palliative care services at all levels of care.^4^

WHO defines palliative care as a person-centred multidimensional and interdisciplinary approach to care, to improve quality at the end of life and the well-being of all patients and their family members facing issues associated with life-limiting illnesses. WHO’s definition stresses that palliative care includes physical, psychosocial and existential dimensions and should, when needed, be provided in all care places and to all patients in need of such care, and can be provided at all care levels, as general palliative care in, for example, hospitals and nursing homes, and as specialised palliative care in specialised care facilities, such as hospices.^5^ The International Association for Hospice and Palliative Care (IAHPC) published a definition of palliative care in 2019. The IAHPC’s view is that the WHO definition limits palliative care to applying only to the problems related to life-threatening illness, rather than to patients’ needs. The IAHPC definition includes people of all ages with severe illness and care given at the end of life in all care places and stresses that access to specialised palliative care should be available for all those in need. Hence, IAHPC’s definition changes the focus from diagnosis and life-threatening illness to the suffering of people with severe illness.^6^ Key elements of palliative care, besides symptom relief and relief of suffering, are support for family members, timely communication and shared decision-making.^7^

Palliative care started to substantially improve care for people with cancer in the 1960s within the hospice movement and has since moved towards the inclusion of people with other chronic diseases.^8^ Hence, palliative care was initially meant to be introduced late in the illness trajectory, with a clear move from curative treatment to palliation. Over time, with the expansion of palliative care to also be relevant for people with all types of chronic and incurable conditions with potential palliative care needs, WHO’s definition of palliative care has come to advocate an early integration of palliative care as well as its continuation throughout the whole illness trajectory. The point at which palliative care is introduced has been shown to depend on the care organisation and existing guidelines.^9^

Healthcare policy should drive the direction of strategies and action to improve care, prevent illness and relieve suffering. Healthcare policy can be established through, for example, laws, clinical guidelines or simply guiding principles.^10^ International studies about the inclusion of palliative care in policies have focused on the inclusion of palliative care in national health policies^11,12^ or, more specifically, in policies of older people,^13^ all concluding that the inclusion of palliative care in policies is sparse. It has been suggested that palliative care policies are essential for informing and guiding the provision of palliative care.^14^ The Swedish healthcare system is governed by democratically elected politicians and is divided into three levels: the state, regions and municipalities. The state establishes the overall political agenda through legislation and is responsible for the development of regulations and guidelines through the National Board of Health and Welfare (NBHW).^15^ Sweden has 21 independent regions which collaborate with municipalities in a joint organisation – The Swedish Association of Local Authorities and Regions (SALAR).^16^ Following the proposals of a report in 2009, which showed large regional differences in cancer care, six Regional Cancer Centres (RCC) were established, to develop cancer care according to the intentions of the national cancer strategy and the EU’s cancer plan.^17^ Quality indicators for palliative care in Swedish policy have been investigated,^18^ as has the historical development of palliative care policy narratives in Sweden, suggesting that the increase in policy documents produced by the Swedish welfare state illustrates that death and dying have become matters of public concern.^19^

Approximately 90,000 people die each year in Sweden – mainly from circulatory diseases and cancer diseases. Of this total, it has been estimated that 80% have potential palliative care needs.^20^ The care for severe chronic conditions and palliative care in Sweden is considered high quality and well developed.^21^ However, there are inequalities in care at the end of life in Sweden for different severe chronic conditions, particularly cancer diseases, despite the development and implementation of palliative care policy.^22^ Overall palliative care needs are expected to increase over time and the inclusion of palliative care in policy has been encouraged internationally. It is therefore paramount to examine how palliative care is included in policy documents in Sweden. A documentary analysis can provide insights into policy and any gaps or inconsistencies in the policy documents for the care of severe chronic conditions with potential palliative care needs. Therefore, this study aimed to examine how palliative care is included in national disease-specific policy documents for adults with chronic conditions, cancer and non-cancer, involving potential palliative care needs.

## Methods

A document analysis of Swedish healthcare policy documents for disease-specific severe chronic conditions, cancer and non-cancer, with potential palliative care needs, was performed in line with Dalglish et al.^23^ This approach involves four consecutive steps: readying the material to gain an overview; reading to identify relevant text and extract data; analysing the body of documents and extracted text segments; and distilling the findings.^23^ For the analysis, our starting point was to identify key elements – namely, the timing of the introduction of palliative care and definitions of palliative care – as described in the background section above. The reporting of this study conforms to the Standards for Reporting Qualitative Research (SRQR) statement^24^ (Supplemental File 1).

### Readying the materials – setting parameters for the study

For the analysis of national disease-specific policy documents, we decided to include all official national policy documents that were valid in 2023 for diagnoses with potential palliative care, based on Murtagh et al.’s^1^ model for diagnoses with potential palliative care needs. The focus of this study was policy documents for adults (⩾18 years old) with cancer and non-cancer diseases with potential palliative care needs.

### National framework for policy production

In Sweden, the development of national care programmes for different cancer diagnoses has since 2009 been established by RCC. These documents are designed by professionals with clinical and research expertise in cooperation with patient representatives and address various aspects of care and treatment for the diagnosis in question. It is mandatory to refer to the national care programme for palliative care, but the authors also have the option to describe palliative care in more detail related to the specific diagnosis.

The NBHW has a long tradition of publishing guidelines for different diagnoses, focusing on management and giving recommendations for treatment based on the state of evidence and established quality indicators. National care programmes for non-cancer conditions, on the other hand, were not introduced until 2020 and have been developed in a similar way as the national cancer care programme, but by SALAR. Additionally, SALAR is responsible for the development of national standardised care processes for various conditions. National standardised care processes for cancer are the responsibility of RCC.

In addition to these disease-specific documents, there is a Swedish national guideline for palliative care published in 2013^18^ and a ‘National knowledge support base for good palliative care at the end of life’, which frames palliative care as care at the very end of life^19^ (both from NBHW), as well as a national care programme for palliative care from RCC.^20^ There is also a Swedish adaptation of WHO’s definition of palliative care, ‘the four cornerstones’, which describes how palliative care is based on symptom relief, teamwork, communication and relationships, as well as family support.^20^ Key elements of palliative care from these documents are of interest for our analysis, for example, the importance of end-of-life conversations when the focus of care changes to a palliative focus is emphasised in the national care programme on palliative care. The NBHW, however, defines an end-of-life conversation as a conversation about care transitions to end-of-life care.^19^

### Document searches

The documents were found mainly via searches on websites of the Swedish government, NBHW, SALAR and RCC. Through the searches, four types of healthcare policies were identified: national overarching strategies, national care programmes, national standardised care processes and national guidelines (Table 1). Initially, a search framework was developed from descriptions of palliative care, key elements and terms described in both the literature^5–7^ and in the Swedish national quality indicators,^25^ described above in the background. One of the researchers reviewed the literature and developed a search framework, which was discussed several times with the research group, and adjusted during the search process to ensure all relevant key elements and terms were included. The search framework consisted of the following terms: Palliative, End-of-life care/Care at the end of life, Terminal care, End-of-life conversation, Family members/Relatives, Support, Death/Dying, Pain, Symptom relief, Suffering, Existential, Quality of life. The overarching analytical questions that were established to guide the analysis were related to how palliative care was included in the documents, that is, whether, and to what extent, palliative care was included, as well as details regarding palliative care.

**Table 1.** Policy documents included in the analysis (*N* = 96).

Type of document	Individuals/entities responsible for development and publication	Number of documents identified
National strategies	Developed and published by the Swedish government	*N* = 4: all four for cancer
National care programmes	Developed by healthcare professionals and patient representatives. National care programmes for cancer are reviewed and published by RCC. National care programmes for non-cancer conditions are reviewed and published by The SALAR	*N* = 49: 47 for cancer and two for severe chronic conditions
National standardised care processes	Developed by representatives of the affected healthcare professionals and patients. National standardised care processes for cancer are reviewed and published by RCC. National standardised care processes for non-cancer conditions are reviewed and published by SALAR	*N* = 36: 30 for cancer and 6 for severe chronic conditions
National guidelines	National guidelines for non-cancer conditions are reviewed and published by NBHW	*N* = 6: all for severe chronic illness

NBHW, National Board of Health and Welfare; RCC, Regional Cancer Centres; SALAR, Swedish Association of Local Authorities and Regions.

### Data extraction

A search for the terms in the developed search framework, based on the guiding questions, was performed to identify and extract text segments about palliative care. Two of the researchers separately searched through all the identified documents for the terms in the search framework, using the search function in Adobe Reader. While data were extracted, the documents were read for overall meaning and the researchers took notes throughout the extraction process.

### Analysis

First, a descriptive overview of all the policy documents was achieved by mapping the topics and formulations pertaining to palliative care. This overview was followed by a compilation of all the searches of all identified documents. An analysis of larger extracts of text segments regarding palliative care and key elements that were identified was conducted focusing on identifying underlying meanings and gaps in the text to understand how palliative care was described. Furthermore, the analysis entailed exploring whether there was a connection to the specific diagnosis; how palliative care was defined or conceptualised, for example, by a commonly accepted definition; and whether key elements of palliative care were described – and with what terms, for example, through synonyms or metaphors not identified in the initial data extraction. The researchers wrote memos throughout the analysis process, which were used when distilling the results. The analysis also included analysing the documents as a whole with regard to the study aim and the key elements searched for, by examining how palliative care and key elements were included, and along what axis, that is, the extent of inclusion of palliative care.^23^ Two of the researchers performed the analysis above separately and then overviewed and discussed the text segments in which palliative care and key elements had been identified.

### Distilling – conceptualisation

Finally, the views of palliative care identified in the documents studied were distilled into overarching conceptualisations of palliative care.

## Results

In total, 96 documents were identified and examined for how palliative care was included – 85 documents for cancer and 11 for severe chronic conditions, with potential palliative care needs. Of all the documents, 43 documents for different cancer types and 10 documents concerning severe non-cancer chronic conditions included palliative care formulations feasible for further analysis. The remaining documents did not include any substantial text about palliative care that was possible to analyse.

The extent to which palliative care was included in the documents differed greatly. All the analysed documents contained the word palliative. However, the scope of the conceptualisation of palliative care in the text segments varied, from none at all to quite substantial chapters dedicated solely to palliative care, mainly in the national cancer care programmes and the national cancer strategies. Some documents did not specify palliative care at all or establish what palliative care could refer to for patients with the specific diagnosis in question, with a reference to palliative care guidelines being the only mention of palliative care in the documents. The use of the word palliative was mainly seen in the context of palliative treatments, such as palliative oncological care or medicine, not as an overarching approach to care. However, there were examples in which recognised definitions of palliative care were included. All care programmes for cancer care referred to the national care programme for palliative care (as expected, given the instruction from RCC to include this reference in all cancer care programmes). References to the national palliative care programme or other national guidelines specific for palliative care varied in the documents for severe non-cancer chronic conditions – some referred to the NBHW’s national guidance for palliative care at the end of life,^26^ while few referred to the national care programme for palliative care.^27^

Conceptualisations of palliative care are presented below, and exemplary excerpts from policy documents are in Table 2.

**Table 2.** Examples of excerpts from policy documents of palliative care conceptualisations and key elements.

Conceptualisations of palliative care	Examples of text excerpts	Policy document
Defined through authorities’ definitions	1. Palliative care is based on an approach that aims to improve the quality of life for the patient by preventing and alleviating suffering. . . 2. The palliative approach is based on the physical, psychological, social and existential dimensions of life and also includes support for the family	National care programme for myeloproliferative neoplasia National care programme for bone and soft tissue sarcoma in extremities and torso
Care of dying persons	3. When a dementia disease has progressed to later and more severe stages, it may be appropriate to switch to palliative care	National care programme for dementia
Integrated with disease-specific care and treatment	4. Palliative interventions can advantageously be integrated with the cancer-specific treatment already early in the course if the patient has cancer symptoms 5. Early integration, that is, that palliative care starts and is carried out in parallel with active treatment in the case of the uncertain prognosis that advanced cirrhosis entails. Even for patients where the treatment has a curative intention, for example, on the waiting list for a liver transplant, a palliative approach should be used	National care programme for breast cancer National care programme for liver cirrhosis
Limited to disease-specific medical treatments	6. In some patients, supportive treatment without chemotherapy may be the wisest palliative strategy. In other patients, the best palliation is achieved through a combination of good supportive treatment and low-intensity symptom-relieving cytostatic treatment or radiotherapy	National care programme for mantle cell lymphoma
Elaboration of key elements of palliative care		
Specialised palliative care	7. Patients in the palliative phase with complex symptoms or whose life situation entails special needs should be cared for by personnel with special knowledge and competence in palliative care, for example, within a specialised palliative practice 8. Specialists in palliative medicine and nurses in palliative care can support seriously ill patients and affected relatives by improving symptom control, reducing anxiety and creating the conditions for care where the patient and relatives wish	National care programme for acute myeloid leukaemia National care programme for malignant melanoma and squamous cell carcinoma
Multidisciplinary teamwork	9. Several professions need to be involved. . .to coordinate medical, palliative, existential and social care	National care programme for liver cirrhosis
End-of-life conversation	10. For very seriously ill patients, an end-of-life conversation, preferably together with family, can lead to a decision to transfer to palliative care 11. In the transition between the late palliative phase and the end of life, an end-of-life conversation must be held	National guideline for stroke/TIA National care programme for biliary cancer

TIA: transient ischaemic attack.

### Palliative care is defined through authorities’ definitions

When describing the aim of and approach to palliative care, WHO’s definition of palliative care^5^ was a common reference, with variations in how detailed and specific the descriptions were. Some of the documents reproduced the whole definition but did not include any clear adaptation or problematisation of what may be involved in palliative care for the specific diagnosis. Other documents included both a full definition of palliative care and an adaptation of relevant interventions for the specific diagnosis, including treatment of disease-specific symptoms and complications. In other documents, only parts of the definition could be traced, focusing on symptom relief and/or quality of life (Table 2, excerpt 1). In some documents, the focus was also on psychological, social and existential dimensions of palliative care, with a holistic view described as physical, psychological, social and existential aspects. For example, in the national care programme for bone and soft tissue sarcoma in extremities and torso, a palliative approach was described based on physical, psychological, social and existential dimensions of life (Table 2, excerpt 2).

### Palliative care as care of dying persons

Death and dying were scarcely mentioned in the documents, except in the context of prognosis or cause of death. Palliative care was thus seen as relevant only during the last period of life – when disease-specific treatment had been exhausted and had ended – and not as an integrated part of the care and treatment during a longer course of a progressive severe chronic condition (both cancer and non-cancer). This view of palliative care as care for the dying was prominent in the documents studied. In these documents, palliative care as care for dying persons appeared as something that replaces disease-specific care and treatment, thus reflecting a dichotomous approach (Table 2, excerpt 3).

### Palliative care integrated with disease-specific care and treatment

There were also examples of national care programmes presenting palliative care as being integrated with – or offered in parallel with – disease-specific treatments and care, thus representing a radically different view from the dichotomous approach above. Particularly in cancer care programmes for diagnoses with relatively high mortality, the importance of an early integrated palliative approach and palliative care treatments alongside the disease-specific treatment was underlined (Table 2, excerpt 4). In contrast to this, however, there were also national care programmes for cancer diagnoses with a high mortality rate that did not include palliative care at all, apart from guidance for anti-tumoural treatments. Nevertheless, both the national care programme for chronic kidney disease and liver cirrhosis highlighted palliative care as integrated with disease-specific efforts. The liver cirrhosis programme also emphasised that a palliative approach with supportive and symptom-relieving treatment and care could be beneficial for patients awaiting transplantation (Table 2, excerpt 5).

### Palliative care is limited to disease-specific medical treatments

An additional view that was displayed limited the scope of palliative care to the recommendation of disease-specific medical treatments. This was primarily identified in care programmes for cancer diagnoses that, with regard to palliative care, solely focused on anti-tumoural treatments, such as disease-specific palliative cytostatic treatment, radiation, palliative surgery and targeted drugs (Table 2, excerpt 6). In these documents, palliative care mainly involved slowing down oncological treatments. Furthermore, palliative care described as detailed medical treatment of disease-specific complications at the end of life could be seen in cancer care programmes, for example, in the care programme for vulvar cancer where surgical treatment was suggested when the consequences of the disease became too severe. Another example was in the care programme for ovarian cancer focusing on treatment of the ileus. In policy documents for severe non-cancer chronic conditions, disease-specific medical treatment in the context of palliative care was identified in only one document – NBHW’s guideline for cardiac care – in which the deactivation of implantable cardioverter defibrillators during the end of life was emphasised as important, and this was the only palliative treatment mentioned.

### Elaboration of key elements of palliative care

There were examples in the documents where key elements of palliative care – primarily specialised palliative care, multidisciplinary teamwork and communication including end-of-life conversations with patients and families – were elaborated on. The need for referral to a palliative consultant or to specialised palliative care was addressed in policy documents for cancer care. However, in the policy documents for severe non-cancer chronic conditions, referral to or inclusion of specialised palliative care was not mentioned. How it was addressed varied and it was sometimes only expressed as an indication that contact or referral may be appropriate, while in other cases a fuller description was included. Other documents provided concrete examples of situations in which contact with specialised palliative care should be established, for example, those involving difficult symptoms or a complex life situation – or for patients with a low-performance status where all possibilities for oncological treatment had been exhausted (Table 2, excerpt 7). Furthermore, there were indications in documents that palliative care interventions should only be provided by healthcare professionals specialised in palliative care (Table 2, excerpt 8).

Another key element of palliative care highlighted in some documents, both for cancer and severe non-cancer chronic conditions, was the importance of multidisciplinary teamwork to support the patient and family. For example, the national care programme for liver cirrhosis stressed how several professions need to be involved in the coordination of medical, palliative, existential and social care (Table 2, excerpt 9).

Documents addressed key elements of communication and end-of-life conversation in different ways. There were examples of documents in which communication and conversation were described, but the contextualisation to the end of life and dying was omitted. In other documents, end-of-life conversation was mentioned, but not described in more detail in terms of timing, how it should be conducted or topics for conversation. In other documents, adherence to the NBHW’s definition was seen, with descriptions of an end-of-life conversation as a conversation during the transition to palliative care at the end of life (Table 2, excerpts 10 and 11). Occasionally, in care programmes for cancer, the importance of ongoing conversations with the patient and family during the illness trajectory was highlighted as was the fact that the transition to palliative care can be difficult to identify – hence, the importance of having an ongoing dialogue with the patient and family about the course of the disease and the trajectory. Having repeated end-of-life conversations was recommended along the disease trajectory, perhaps involving an initial conversation when a cure is no longer possible and then a further conversation later when the focus shifts from life extension to quality of life.

## Discussion

This study aimed to examine how palliative care is included in national disease-specific policy documents for adults with chronic conditions, cancer and non-cancer, with potential palliative care needs. The results showed large variations in how palliative care was included in the policy documents, from mentioning the term without explanation to detailed discussion regarding palliative care practice. Variations in how palliative care was included encompassed conceptualisations of palliative care through established definitions, palliative care as care of dying persons and palliative care limited to disease-specific medical treatments or as an integrated care approach, and elaborations on certain key elements of palliative care, such as specialised palliative care and end-of-life conversations. All these conceptualisations and key elements are highly relevant for palliative care. However, a limitation of the conceptualisations identified is that most of the policy documents analysed included only one conceptualisation; for example, either palliative care as care for dying people or palliative care as disease-specific medical treatments. Such conceptualisations are not in line with the international palliative care policy or research evidence. A dichotomic view of palliative care to substitute diagnosis-specific treatment and care leads to the omission of integrating palliative approaches earlier on. This has unfortunate consequences, especially for people with a diagnosis that impacts cognitive function. Here, we underline that we fully agree that including disease-specific medical treatments in these types of policy documents is both relevant and important. It is surprising that not all national disease-specific policy documents include disease-specific palliative medical treatments in addition to specific assessments and interventions adapted to the specific diagnosis (including pathophysiology, treatments, common symptoms and other consequences). In this regard, a palliative approach for long-term progressive conditions^9^ is lacking in the policy documents analysed. Furthermore, limiting policy regarding palliative care to medical treatments highlights the omission of nursing priorities in national healthcare policy documents.^28^

Palliative care conceptualised through established definitions was mainly based on the WHO’s definition of palliative care and the Swedish adaptation of the WHO’s definition (‘the four cornerstones’ of palliative care).^27^ This conceptualisation, which does not include the full definition of palliative care at any point in the policy documents, shows a limited view of palliative care. Early integration of palliative care is not explicitly addressed in the studied policy documents, despite WHO’s emphasis on the importance of this point.^5^ The IAHPC definition of palliative care^6^ is not seen in any of the policy documents, and may further reflect the fact that national discussion about the IAHPC definition has been limited and has involved criticism. Notably, this definition is not included in any type of national palliative care policy document in Sweden. Furthermore, the key elements of palliative care that were focused on in the documents – mainly specialised palliative care and to some extent multidisciplinary teamwork and end-of-life conversations – largely concerned the very end of life, again presenting a view of palliative care as the care of dying persons. The results of this study are similar to those of other international studies in which the inclusion of palliative care in policy has been investigated. Pivodic et al.^13^ studied the inclusion of palliative care in public policy documents on healthcare for older people in 13 countries and found that only a fifth of the documents explicitly mentioned palliative care. The authors concluded that the inclusion of key elements of palliative care is instead described within central elements of other types of care, for example, rehabilitation or long-term care. This was also the case in the present study in which certain elements, such as communication and family support, were described in connection to cancer care or rehabilitation rather than palliative care. Pivodic et al.^13^ state that healthcare policies need revision to further include reference to palliative care and dying and to ensure that they are linked to existing national or regional palliative care strategies.

The ongoing move towards recognising the relevance of palliative care to include diagnoses other than cancer^9^ is reflected in the results of this study, although they also highlight that the inclusion of palliative care is not as explicit in policy documents for non-cancer chronic conditions as it is in documents for cancer diseases. The results of this study also showed that, even in documents for cancer diseases, the inclusion of palliative care is limited. Several studies have pointed to how access to palliative care depends on the type of disease. For example, people with diseases other than cancer, such as chronic pulmonary lung disease, heart disease and dementia, have poorer access to palliative care than those with cancer,^22,29^ resulting in less adequate symptom control and less communication about end-of-life issues.^30–32^ Cancer diseases may be perceived as having a clearer illness trajectory than other chronic diseases or in the case of older persons.^33,34^ It may be that such perceptions could be connected to differing views of palliative care related to different severe chronic conditions. Additionally, the stereotyped views of professionals contributing to the development of the policies may impact how palliative care is included. Variations in the inclusion of palliative care in policy documents which are frequently used by the clinical professions may contribute to the prevailing inequalities in palliative care in care for severe chronic conditions, which ultimately affects patients and their families.

Inequality in palliative care and care at the end of life has been raised internationally,^35,36^ and through Swedish governmental evaluations and investigations ever since the mid-90s, and initiatives have been taken to raise the awareness and inclusion of palliative care, both in policy and care, for example, through the establishment of the six RCC and the development of national care programmes for specific cancer diagnoses. Gao et al.^14^ state that palliative care policies inform and guide the provision of palliative care, for example, healthcare service characteristics – including service type, facilities, workforce types and levels of palliative care. Ågren et al.^19^ concluded that the increase in policy documents produced by the Swedish welfare state illustrates that death and dying have become matters of public concern through increased involvement of the welfare state in defining what palliative care is, identifying problems within these care contexts and offering solutions for such problems. However, they also found that policy narratives display a tension between medicalisation of palliative care on the one hand and the importance of acknowledging the social, psychological and emotional needs of the dying person and family on the other. This tension can be clearly seen in the results of this documentary analysis, in which medicalisation dominates. Ågren et al.^19^ suggest that the tension also highlights the ongoing evolving work to define what palliative care is, which – it is often argued – is key to success in the healthcare and medical spheres. The lack of inclusion of palliative care in the studied policy documents or inclusion mainly focused on disease-specific medical treatments may come down to there being no shared view of palliative care and care at the end of life on the part of decision-makers and policy makers. In this regard, a minimum ‘point-of-departure’ for palliative care would possibly bring about a more equal and consistent inclusion of palliative care in policy documents.

The inclusion of palliative care in the documents that were analysed in the present study has been initiated via a top-down approach, that is, it has been decided on a national level that palliative care is to be included. Whitelaw et al.^37^ suggest an implementation gap between policy and practice, also connected to the approach for policy development being initiated from a ‘catastrophic’ top-down approach in which there is a crisis needing urgent solutions. Policy may be seen as one element in a complex mix involving other parts, particularly higher levels of participation by health service providers and the wider civic society, but bottom-up approaches are often driven by motivated individuals and nongovernmental organisations with potentially limited financial, political and policy influence. The present study revealed a lack of inclusion of palliative care in the policy documents analysed – or inclusion mainly focused on disease-specific medical treatments without elaboration on palliative care. In line with this Clelland et al.^11^ investigated national palliative care strategies, plans, legislation and dedicated government resources worldwide, and concluded that there is a long way to go before palliative care is universally supported by policy that can lead to observable results. They also highlighted the need for greater clarity regarding which actions and indicators can be considered – based on policy – as a response to the populations’ need for palliative care.

## Limitations

This study was limited to publicly available national policies. Unofficial documents were excluded, which could have shed light on the policy development process. There may be other regional or local policies that include palliative care differently. Regional differences in the Swedish healthcare system and differences in procedures for different diseases may impact the way in which policies are developed, shaped and implemented. Furthermore, the policy documents were developed by different actors which may have influenced how palliative care was included and described. In addition, the policies included in this study were written over a time period during which palliative care has continued to evolve, which may also have influenced the focus of the documents and how palliative care was included and described. Paediatric policy documents were excluded since cancer and non-cancer palliative care in children is quite different from that relating to the adult population, and paediatric palliative care in Sweden is organised slightly differently from palliative care in adults.

## Conclusion

There may be large variations in how palliative care is conceptualised in national disease-specific policy documents, as disclosed by this analysis of the Swedish case. Limiting palliative care to disease-specific medical treatments (most commonly palliative oncological treatments) or to the care of dying persons limits its scope in ways that are contrary to current evidence in support of early integrated palliative care. The lack of palliative care recommendations adapted for each specific diagnosis indicates a gap in policy. To promote equal access to palliative care regardless of patients’ diseases or medical conditions, the importance of how palliative care is included in national policy documents needs to be further acknowledged and discussed – with palliative care consistently included in such documents. Widespread, explicit inclusion of palliative care in disease-specific policy documents, such as strategies and guidelines, can also improve the implementation of early palliative care, which has been shown to be beneficial for patients and family members in previous research. Prerequisites for the establishment of palliative care policy targeting specific diseases are a shared view of the concept of palliative care, what it entails and an implementation of policy, although it should be noted that policy alone if not established within the healthcare professions, may have limited potential to bring about significant change.

## Supplemental Material

sj-docx-1-pcr-10.1177_26323524241296145 – Supplemental material for Palliative care in policy documents for adults with cancer and non-cancer diseases with potential palliative care needs: a document analysis Supplemental material, sj-docx-1-pcr-10.1177_26323524241296145 for Palliative care in policy documents for adults with cancer and non-cancer diseases with potential palliative care needs: a document analysis by Anna O’Sullivan, Linnéa Carling, Joakim Öhlén, Stina Nyblom, Anneli Ozanne, Ragnhild Hedman, Carl-Johan Fürst and Cecilia Larsdotter in Palliative Care and Social Practice

sj-docx-2-pcr-10.1177_26323524241296145 – Supplemental material for Palliative care in policy documents for adults with cancer and non-cancer diseases with potential palliative care needs: a document analysis Supplemental material, sj-docx-2-pcr-10.1177_26323524241296145 for Palliative care in policy documents for adults with cancer and non-cancer diseases with potential palliative care needs: a document analysis by Anna O’Sullivan, Linnéa Carling, Joakim Öhlén, Stina Nyblom, Anneli Ozanne, Ragnhild Hedman, Carl-Johan Fürst and Cecilia Larsdotter in Palliative Care and Social Practice

## Declarations

**Ethics approval and consent to participate:** Not applicable.

**Consent for publication:** Not applicable.

**Availability of data and materials:** The data analysed during the current study are publicly available, see Supplemental File 2 available online with a list of web links to all documents included in the study analysis.
