# Supplementary material for: Palliative care in policy documents for adults with cancer and non-cancer diseases with potential palliative care needs: a document analysis
Source: Palliat Care Soc Pract. 2024 Dec 3;18:26323524241296145. doi: 10.1177/26323524241296145 (PMC11615978; doi:10.1177/26323524241296145)
Supplement: sj-docx-2-pcr-10.1177_26323524241296145 – Supplemental material for Palliative care in policy documents for adults with cancer and non-cancer diseases with potential palliative care needs: a document analysis [file sj-docx-2-pcr-10.1177_26323524241296145.docx]

LIST OF WEB LINKS TO ALL DOCUMENTS INCLUDED IN THE ANALYSIS OF TEXT

SEGMENTS ABOUT PALLIATIVE CARE

Cancer

1. Regional Cancer Centres in Sweden - Regionala cancercentrum i samverkan. 2021. *Aggressive B-cell lymphomas - Aggressiva B-cellslymfom*: [Nationellt vårdprogram aggressiva B-cellslymfom - RCC Kunskapsbanken (cancercentrum.se)](https://kunskapsbanken.cancercentrum.se/diagnoser/aggressiva-b-cellslymfom/vardprogram/)
2. Regional Cancer Centres in Sweden - Regionala cancercentrum i samverkan. 2021. *Acute lymphocytic leukemia - Akut lymfatisk leukemi*: [Nationellt vårdprogram ALL (cancercentrum.se)](https://kunskapsbanken.cancercentrum.se/globalassets/cancerdiagnoser/blod-lymfom-myelom/all/vardprogram/nationellt-vardprogram-akut-lymfatisk-leukemi-all.pdf)
3. Regional Cancer Centres in Sweden - Regionala cancercentrum i samverkan. 2021. *Acute myeloid leukemia - Akut myeloisk leukemi (AML)*: [Nationellt vårdprogram AML (cancercentrum.se)](https://kunskapsbanken.cancercentrum.se/globalassets/cancerdiagnoser/blod-lymfom-myelom/aml/vardprogram/nationellt-vardprogram-akut-myeloisk-leukemi-aml.pdf)
4. Regional Cancer Centres in Sweden - Regionala cancercentrum i samverkan. 2020. *Acute oncology -* *Akut onkologi*: [Akut onkologi (cancercentrum.se)](https://kunskapsbanken.cancercentrum.se/globalassets/cancerdiagnoser/akut-onkologi/nationellt-vardprogram-akut-onkologi.pdf)
5. Regional Cancer Centres in Sweden - Regionala cancercentrum i samverkan 2020. *Anal cancer –* *Analcancer*: [Nationellt vårdprogram analcancer (cancercentrum.se)](https://kunskapsbanken.cancercentrum.se/globalassets/cancerdiagnoser/tjock--och-andtarm-anal/vardprogram/nationellt-vardprogram-analcancer.pdf)
6. Regional Cancer Centres in Sweden - Regionala cancercentrum i samverkan. 2020. *Adrenal tumors – Binjuretumörer*: [Nationellt vårdprogram binjuretumörer - RCC Kunskapsbanken (cancercentrum.se)](https://kunskapsbanken.cancercentrum.se/diagnoser/binjuretumorer/vardprogram/)
7. Regional Cancer Centres in Sweden - Regionala cancercentrum i samverkan. 2022. *Breast cancer – Bröstcancer*: [Nationellt vårdprogram bröstcancer (cancercentrum.se)](https://kunskapsbanken.cancercentrum.se/globalassets/cancerdiagnoser/brost/vardprogram/nationellt-vardprogram-brostcancer.pdf)
8. Regional Cancer Centres in Sweden - Regionala cancercentrum i samverkan. 2022. *Abdominal*

*sarcoma Intra-abdominal, retroperitoneal and gynecological soft tissue sarcomas -* *Buksarkom Intraabdominella, retroperitoneala och gynekologiska mjukdelssarkom*: [Nationellt vårdprogram buksarkom - RCC Kunskapsbanken (cancercentrum.se)](https://kunskapsbanken.cancercentrum.se/diagnoser/buksarkom/Vardprogram/)

1. Regional Cancer Centres in Sweden - Regionala cancercentrum i samverkan. 2021. *Pancreatic cancer –* *Bukspottkörtelcancer*:  [Nationellt vårdprogram bukspottkörtelcancer - RCC Kunskapsbanken (cancercentrum.se)](https://kunskapsbanken.cancercentrum.se/diagnoser/bukspottkortelcancer/vardprogram/)
2. Regional Cancer Centres in Sweden - Regionala cancercentrum i samverkan. 2021. *Cancer without known primary tumor -* *Cancer utan känd primärtumör*: [Nationellt vårdprogram cancer utan känd primärtumör, CUP - RCC Kunskapsbanken (cancercentrum.se)](https://kunskapsbanken.cancercentrum.se/diagnoser/cancer-utan-kand-primartumor-cup/vardprogram/)
3. Regional Cancer Centres in Sweden - Regionala cancercentrum i samverkan. 2019. *Gallbladder and bile duct cancer - Gallblåse- och gallvägscancer*: [Nationellt vårdprogram gallblåse- och gallvägscancer - RCC Kunskapsbanken (cancercentrum.se)](https://kunskapsbanken.cancercentrum.se/diagnoser/gallblase-och-gallvagscancer/vardprogram/)
4. Regional Cancer Centres in Sweden - Regionala cancercentrum i samverkan. 2020. *Tumors of the brain and spinal cord - Tumörer i hjärna och ryggmärg*: [Nationellt vårdprogram tumörer i hjärna och ryggmärg - RCC Kunskapsbanken (cancercentrum.se)](https://kunskapsbanken.cancercentrum.se/diagnoser/hjarna/vardprogram/)
5. Regional Cancer Centres in Sweden - Regionala cancercentrum i samverkan. 2022. *Hodgkin's lymphoma - Hodgkins lymfom*: [Nationellt vårdprogram Hodgkins lymfom - RCC Kunskapsbanken (cancercentrum.se)](https://kunskapsbanken.cancercentrum.se/diagnoser/hodgkins-lymfom/vardprogram/)
6. Regional Cancer Centres in Sweden - Regionala cancercentrum i samverkan. 2022. *Skin lymphoma - Hudlymfom*: [Nationellt vårdprogram hudlymfom - RCC Kunskapsbanken (cancercentrum.se)](https://kunskapsbanken.cancercentrum.se/diagnoser/hudlymfom/vardprogram/)
7. Regional Cancer Centres in Sweden - Regionala cancercentrum i samverkan. 2022. *Head and neck cancer -* *Huvud- och halscancer*: [Nationellt vårdprogram huvud- och halscancer - RCC Kunskapsbanken (cancercentrum.se)](https://kunskapsbanken.cancercentrum.se/diagnoser/huvud-och-halscancer/vardprogram/)
8. Regional Cancer Centres in Sweden - Regionala cancercentrum i samverkan. 2021. *Pituitary tumors –* *Hypofystumörer*: [Nationellt vårdprogram Hypofystumörer (cancercentrum.se)](https://kunskapsbanken.cancercentrum.se/globalassets/cancerdiagnoser/hypofystumorer/vardprogram/nationellt-vardprogram-hypofystumorer.pdf)
9. Regional Cancer Centres in Sweden - Regionala cancercentrum i samverkan. 2020. *Indolent B-cell lymphoma and hair cell leukemia - Indolenta B-cellslymfom och hårcellsleukemi*: [Nationellt vårdprogram för indolenta B-cellslymfom och hårcellsleukemi (cancercentrum.se)](https://kunskapsbanken.cancercentrum.se/globalassets/cancerdiagnoser/blod-lymfom-myelom/lymfom/vardprogram/nationellt-vardprogram-indolenta-b-cellslymfom.pdf)
10. Regional Cancer Centres in Sweden - Regionala cancercentrum i samverkan. 2022. *Chronic lymphocytic leukemia - Kronisk lymfatisk leukemi (KLL)*: [Nationellt vårdprogram KLL (cancercentrum.se)](https://kunskapsbanken.cancercentrum.se/globalassets/cancerdiagnoser/blod-lymfom-myelom/kll/vardprogram/nationellt-vardprogram-kronisk-lymfatisk-leukemi-kll.pdf)
11. Regional Cancer Centres in Sweden - Regionala cancercentrum i samverkan. 2019. *Chronic myeloid leukemia -* *Kronisk myeloisk leukemi*: [Nationellt vårdprogram för KML (cancercentrum.se)](https://kunskapsbanken.cancercentrum.se/globalassets/cancerdiagnoser/blod-lymfom-myelom/kml/nationellt-vardprogram-kronisk-myeloisk-leukemi-kml.pdf)
12. Regional Cancer Centres in Sweden - Regionala cancercentrum i samverkan. 2022. *Hepatocellular carcinoma – Levercellscancer*: [Nationellt vårdprogram för levercellscancer, HCC (cancercentrum.se)](https://kunskapsbanken.cancercentrum.se/globalassets/cancerdiagnoser/lever-och-galla/vardprogram/nationellt-vardprogram-levercellscancer.pdf)
13. Regional Cancer Centres in Sweden - Regionala cancercentrum i samverkan. 2020. *Cervical cancer and vaginal cancer -* *Livmoderhals cancer och vaginalcancer*: [Nationellt vårdprogram livmoderhalscancer (cancercentrum.se)](https://kunskapsbanken.cancercentrum.se/globalassets/cancerdiagnoser/gynekologi/livmoderhalscancer/varprogram/nationellt-vardprogram-livmoderhalscancer-cervixcancer-vaginalcancer.pdf)
14. Regional Cancer Centres in Sweden - Regionala cancercentrum i samverkan. 2021. *Uterine body cancer (endometrial/corpus cancer) - Livmoder kroppscancer (endometrie-/corpuscancer)*: [Nationellt vårdprogram livmoderhalscancer (cancercentrum.se)](https://kunskapsbanken.cancercentrum.se/globalassets/cancerdiagnoser/gynekologi/livmoderkroppscancer/vardprogram/nationellt-vardprogram-endometriecancer.pdf)
15. Regional Cancer Centres in Sweden - Regionala cancercentrum i samverkan. 2022. *Lung cancer –* *Lungcancer*: [Nationellt vårdprogram lungcancer (cancercentrum.se)](https://kunskapsbanken.cancercentrum.se/globalassets/cancerdiagnoser/lunga-och-lungsack/vardprogram/nationellt-vardprogram-lungcancer.pdf)
16. Regional Cancer Centres in Sweden - Regionala cancercentrum i samverkan. 2021. *Mantle cell lymphoma – Mantelcellslymfom*: [Nationellt vårdprogram mantelcellslymfom (cancercentrum.se)](https://kunskapsbanken.cancercentrum.se/globalassets/cancerdiagnoser/blod-lymfom-myelom/lymfom/vardprogram/nationellt-vardprogram-mantelcellslymfom.pdf)
17. Regional Cancer Centres in Sweden - Regionala cancercentrum i samverkan. 2022. *Esophageal and stomach cancer -* *Matstrups- och magsäckscancer*: [Nationellt vårdprogram för matstrups- och magsäckscancer (cancercentrum.se)](https://kunskapsbanken.cancercentrum.se/globalassets/cancerdiagnoser/matstrupe-och-magsack/vardprogram/nationellt-vardprogram-matstrups-magsackscancer.pdf)
18. Regional Cancer Centres in Sweden - Regionala cancercentrum i samverkan. 2022. *Merkel cell carcinoma – Merkelcellscancer*: [Nationellt vårdprogram merkelcellscancer (cancercentrum.se)](https://kunskapsbanken.cancercentrum.se/globalassets/cancerdiagnoser/hud/merkelcellscancer/vardprogram/nationellt-vardprogram-merkelcellscancer.pdf)
19. Regional Cancer Centres in Sweden - Regionala cancercentrum i samverkan. 2022. *Myeloma –* *Myelom*: [Nationellt vårdprogram för myelom (cancercentrum.se)](https://kunskapsbanken.cancercentrum.se/globalassets/cancerdiagnoser/blod-lymfom-myelom/myelom/vardprogram/nationellt-vardprogram-myelom.pdf)
20. Regional Cancer Centres in Sweden - Regionala cancercentrum i samverkan. 2022. *Myeloproliferative neoplasia -* *Myeloproliferativ neoplasi (MPN)*: [Nationellt vårdprogram MPN (cancercentrum.se)](https://kunskapsbanken.cancercentrum.se/globalassets/cancerdiagnoser/blod-lymfom-myelom/mpn/vardprogram/nationellt-vardprogram-mpn.pdf)
21. Regional Cancer Centres in Sweden - Regionala cancercentrum i samverkan. 2021. *Abdominal neuroendocrine tumors -* *Neuroendokrina buktumörer (GEP-NET)*: [Nationellt vårdprogram för neuroendokrina buktumörer (cancercentrum.se)](https://kunskapsbanken.cancercentrum.se/globalassets/cancerdiagnoser/neuroendokrina-buktumorer/vardprogram/nationellt-vardprogram-neuroendokrina-buktumorer-gep-net.pdf)
22. Regional Cancer Centres in Sweden - Regionala cancercentrum i samverkan. 2022. *Kidney cancer –* *Njurcancer*: [Nationellt vårdprogram Njurcancer (cancercentrum.se)](https://kunskapsbanken.cancercentrum.se/globalassets/cancerdiagnoser/urinvagar/njurcancer/nationellt-vardprogram-njurcancer.pdf)
23. Regional Cancer Centres in Sweden - Regionala cancercentrum i samverkan. 2019. *Penile cancer –* *Peniscancer*: [Nationellt vårdprogram Peniscancer 2019 (cancercentrum.se)](https://kunskapsbanken.cancercentrum.se/globalassets/cancerdiagnoser/peniscancer/vardprogram/nationellt-vardprogram-peniscancer.pdf)
24. Regional Cancer Centres in Sweden - Regionala cancercentrum i samverkan. 2022. *Prostate cancer – Prostatacancer*: [Nationellt vårdprogram för prostatacancer (cancercentrum.se)](https://kunskapsbanken.cancercentrum.se/globalassets/cancerdiagnoser/prostatacancer/vardprogram/nationellt-vardprogram-prostatacancer.pdf)
25. Regional Cancer Centres in Sweden - Regionala cancercentrum i samverkan. 2022. *Bone and soft tissue sarcoma in extremities and trunk wall - Skelett- och mjukdelssarkom i extremiteter och bålvägg*: [Nationellt vårdprogram Skelett- och mjukdelssarkom (cancercentrum.se)](https://kunskapsbanken.cancercentrum.se/globalassets/cancerdiagnoser/sarkom/vardprogram/nationellt-vardprogram-skelett-mjukdelssarkom.pdf)
26. Regional Cancer Centres in Sweden - Regionala cancercentrum i samverkan. 2021. *Squamous cell carcinoma of the skin - Skivepitelcancer i huden*: [Nationellt vårdprogram för skivepitelcancer i huden (cancercentrum.se)](https://kunskapsbanken.cancercentrum.se/globalassets/cancerdiagnoser/hud/skivepitelcancer-i-huden/vardprogram/nationellt-vardprogram-skivepitelcancer-i-huden.pdf)
27. Regional Cancer Centres in Sweden - Regionala cancercentrum i samverkan. 2021. *Thyroid cancer – Sköldkörtelcancer*: [Nationellt vårdprogram sköldkörtelcancer (cancercentrum.se)](https://kunskapsbanken.cancercentrum.se/globalassets/cancerdiagnoser/skoldkortel/vardprogram/nationellt-vardprogram-skoldkortelcancer.pdf)
28. Regional Cancer Centres in Sweden - Regionala cancercentrum i samverkan. 2021. *T-cell lymphoma -* *T-cellslymfom*: [Nationellt vårdprogram T-cellslymfom (cancercentrum.se)](https://kunskapsbanken.cancercentrum.se/globalassets/cancerdiagnoser/blod-lymfom-myelom/lymfom/vardprogram/nationellt-vardprogram-t-cellslymfom.pdf)
29. Regionala cancercentrum i samverkan. 2021. *Tjock- och ändtarmscancer.* [Nationellt vårdprogram tjock- och ändtarmscancer (cancercentrum.se)](https://kunskapsbanken.cancercentrum.se/globalassets/cancerdiagnoser/tjock--och-andtarm-anal/vardprogram/nationellt-vardprogram-tjock-andtarmscancer.pdf)
30. Regional Cancer Centres in Sweden - Regionala cancercentrum i samverkan. 2016. *Gestational trophoblastic disorders -* *Gestationella trofoblastsjukdomar (GTD)*: [Microsoft Word - NVP Trofoblastsjd_160916 SLUTVERSION.docx (cancercentrum.se)](https://kunskapsbanken.cancercentrum.se/globalassets/cancerdiagnoser/gynekologi/trofoblast/nvp-trofoblast.pdf)
31. Regional Cancer Centres in Sweden - Regionala cancercentrum i samverkan. 2021. *Cancer of the bladder, renal pelvis, ureters and urethra - Cancer i urinblåsa, njurbäcken, urinledare och urinrör*: [Nationellt vårdprogram för cancer i urinblåsan (cancercentrum.se)](https://kunskapsbanken.cancercentrum.se/globalassets/cancerdiagnoser/urinvagar/urinblase--och-urinrorscancer/vardprogram/nationellt-vardprogram-urinblase-och-urinvagscancer.pdf)
32. Regional Cancer Centres in Sweden - Regionala cancercentrum i samverkan. 2019. *Vulvar cancer –* *Vulvacancer*: [Nationellt vårdprogram för vulvacancer (cancercentrum.se)](https://kunskapsbanken.cancercentrum.se/globalassets/cancerdiagnoser/gynekologi/vulva/vardprogram/nationellt-vardprogram-vulvacancer.pdf)
33. Regional Cancer Centres in Sweden - Regionala cancercentrum i samverkan. 2021. *Waldenström's macroglobulinemia -* *Waldenströms makroglobulinemi*: [Nationellt vårdprogram Waldenströms makroglobulinemi (cancercentrum.se)](https://kunskapsbanken.cancercentrum.se/globalassets/cancerdiagnoser/blod-lymfom-myelom/lymfom/vardprogram/nationellt-vardprogram-waldenstroms-makroglobulinemi.pdf)
34. Regional Cancer Centres in Sweden - Regionala cancercentrum i samverkan. 2022. *Ovarian cancer with epithelial histology - Äggstockscancer med epitelial histologi*: [Nationellt vårdprogram för äggstockscancer med epitelial histologi (cancercentrum.se)](https://kunskapsbanken.cancercentrum.se/globalassets/cancerdiagnoser/gynekologi/aggstockscancer/nationellt-vardprogram-aggstockscancer.pdf)
35. Regional Cancer Centres in Sweden - Regionala cancercentrum i samverkan. 2018. *Non-epithelial ovarian tumors - Icke-epiteliala äggstockstumörer*: [Nationellt vårdprogram mall (cancercentrum.se)](https://kunskapsbanken.cancercentrum.se/globalassets/cancerdiagnoser/gynekologi/aggstockscancer/nationellt-vardprogram-icke-epiteliala-aggstockstumorer.pdf)

Non-cancer

- 1. Swedish Regions in collaboration - Sveriges regioner i samverkan. 2020. *A personcentred and coordinated care process for Chronic obstructive pulmonary disease (COPD) - Personcentrerat och sammanhållet vårdförlopp Kroniskt obstruktiv lungsjukdom (KOL):* [Kroniskt obstruktiv lungsjukdom (KOL) - Nationellt kliniskt kunskapsstöd (nationelltklinisktkunskapsstod.se)](https://www.nationelltklinisktkunskapsstod.se/kunskapsstod/vardforlopp/kroniskt-obstruktiv-lungsjukdom-kol/)
  2. Swedish Regions in collaboration - Sveriges regioner i samverkan. 2020. *A personcentred and coordinated care process for Stroke and TIA – early measures - Personcentrerat och sammanhållet vårdförlopp*

*Stroke och TIA - tidiga insatser:* [Stroke och TIA - Nationellt kliniskt kunskapsstöd (nationelltklinisktkunskapsstod.se)](https://www.nationelltklinisktkunskapsstod.se/kunskapsstod/vardforlopp/stroke-och-tia--tidiga-insatser-och-vard/)

- 1. Swedish Regions in collaboration - Sveriges regioner i samverkan. 2022. Swedish Regions in collaboration (2020). *A personcentred and coordinated care process for Stroke and TIA, continuing care and rehabilitation - Personcentrerat och sammanhållet vårdförlopp Stroke och TIA, uppföljning*: [Stroke och TIA - fortsatt vård och rehabilitering - Nationellt kliniskt kunskapsstöd](https://www.nationelltklinisktkunskapsstod.se/kunskapsstod/vardforlopp/stroke-och-tia---fortsatt-vard-och-rehabilitering/)

[(nationelltklinisktkunskapsstod.se)](https://www.nationelltklinisktkunskapsstod.se/kunskapsstod/vardforlopp/stroke-och-tia---fortsatt-vard-och-rehabilitering/)

- 1. National Board of Health and Welfare - Socialstyrelsen. 2017. *National guidelines for care and support in dementia. Guidance for management and leadership - Nationella riktlinjer för vård och omsorg vid demenssjukdom. Stöd för styrning och ledning*: [Nationella riktlinjer för vård och omsorg vid demenssjukdom – Stöd för styrning och ledning (socialstyrelsen.se)](https://www.socialstyrelsen.se/globalassets/sharepoint-dokument/artikelkatalog/nationella-riktlinjer/2017-12-2.pdf)
  2. National Board of Health and Welfare - Socialstyrelsen. 2018. *National guidelines heart diseases.*

*Guidance for management and leadership - Nationella riktlinjer för hjärtsjukvård. Stöd för styrning och ledning*:

[Nationella riktlinjer för hjärtsjukvård - Stöd för styrning och ledning (socialstyrelsen.se)](https://www.socialstyrelsen.se/globalassets/sharepoint-dokument/artikelkatalog/nationella-riktlinjer/2018-6-28.pdf)

- 1. National Board of Health and Welfare - Socialstyrelsen. 2020. *National guidelines for care in asthma and COPD. Guidance for management and leadership - Nationella riktlinjer för vård vid astma och KOL. Stöd för styrning och ledning*: [Nationella riktlinjer för vård vid astma och KOL - Stöd för styrning och ledning (socialstyrelsen.se)](https://www.socialstyrelsen.se/globalassets/sharepoint-dokument/artikelkatalog/nationella-riktlinjer/2020-12-7135.pdf)
  2. National Board of Health and Welfare - Socialstyrelsen. 2020. *National guidelines for care in stroke.*

*Guidance for management and leadership - Nationella riktlinjer för vård vid stroke. Stöd för styrning och ledning*: [Nationella riktlinjer för vård vid stroke (socialstyrelsen.se)](https://www.socialstyrelsen.se/globalassets/sharepoint-dokument/artikelkatalog/nationella-riktlinjer/2020-1-6545.pdf)

- 1. National Board of Health and Welfare - Socialstyrelsen. 2022. *National guidelines for care in multiple sclerosis and Parkinsons disease. Guidance for management and leadership - Nationella riktlinjer för vård vid multipel skleros och Parkinsons sjukdom*: [Nationella riktlinjer för vård vid multipel skleros och Parkinsons sjukdom (socialstyrelsen.se)](https://www.socialstyrelsen.se/globalassets/sharepoint-dokument/artikelkatalog/nationella-riktlinjer/2022-11-8202.pdf)
  2. National program group for gastrointestinal diseases - Nationellt programområde för magtarmsjukdomar. 2022. *A national care program for liver cirrhosis - Nationellt vårdprogram för levercirros*: [Nationellt vårdprogram för levercirros (nationelltklinisktkunskapsstod.se)](https://www.nationelltklinisktkunskapsstod.se/globalassets/nkk/nationell/media/dokument/kunskapsstod/vardprogram/nationellt-vardprogram-for-levercirros.pdf)
  3. National program group for renal and urological diseases - Nationellt programområde för urinvägssjukdomar. 2022. *A national care programme for chronic kidney diseases - Nationellt vårdprogram för kronisk njursjukdom*: [Nationellt vårdprogram för kronisk njursjukdom](https://d2flujgsl7escs.cloudfront.net/external/Nationellt_vardprogram_for_kronisk_njursjukdom.pdf)
